# Inexpensive protein overexpression driven by the NarL transcription activator protein

**DOI:** 10.1002/bit.28071

**Published:** 2022-03-06

**Authors:** Joanne Hothersall, Sandie Lai, Nan Zhang, Rita E. Godfrey, Patcharawarin Ruanto, Sarah Bischoff, Colin Robinson, Tim W. Overton, Stephen J. W. Busby, Douglas F. Browning

**Affiliations:** ^1^ School of Biosciences, Institute of Microbiology and Infection University of Birmingham Birmingham UK; ^2^ School of Chemical Engineering University of Birmingham Birmingham UK; ^3^ School of Life Sciences University of Warwick, Gibbet Hill Campus Coventry UK; ^4^ School of Biosciences University of Kent Canterbury UK; ^5^ College of Health & Life Sciences Aston University, Aston Triangle Birmingham UK

**Keywords:** biopharmaceuticals, *Escherichia coli*, recombinant protein production, transcription regulation

## Abstract

Most *Escherichia coli* overexpression vectors used for recombinant protein production (RPP) depend on organic inducers, for example, sugars or simple conjugates. However, these can be expensive and, sometimes, chemically unstable. To simplify this and to cut the cost of RPP, we have developed vectors controlled by the *Escherichia coli* nitrate‐responsive NarL transcription activator protein, which use nitrate, a cheap, stable, and abundant inorganic ion, to induce high‐level controlled RPP. We show that target proteins, such as green fluorescent protein, human growth hormone, and single‐chain variable region antibody fragments can be expressed to high levels using our promoter systems. As nitrate levels are high in many commercial fertilizers, we demonstrate that controlled RPP can be achieved using readily available and inexpensive garden products.

## Graphical abstract

Most *Escherichia coli* overexpression vectors used for recombinant protein production (RPP) depend on organic inducers, such as IPTG, which can be expensive and chemically unstable. To cut the cost of RPP, Hothersall and co‐workers have developed new expression systems, which use nitrate, a cheap, stable and abundant inorganic ion, to induce high‐level RPP. As nitrate levels are high in many commercial fertilizers, they demonstrate that controlled RPP can even be achieved using inexpensive garden products purchased from any local store.

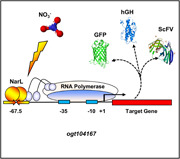

## 1 INTRODUCTION

Many bacterial promoters are highly regulated, and, since the 1970s, biotechnologists have exploited this to control overexpression of foreign proteins in hosts such as *Escherichia coli* (Nora et al., 2019; Tungekar et al., 2021). Most currently used overexpression vectors depend on organic inducers, for example, sugars or simple conjugates, such as IPTG (isopropyl β‐d‐1‐thiogalactopyranoside). However, IPTG is costly, and is unstable, requiring a refrigerated supply chain. Our aim was to construct a new expression system that permits high‐level expression of biopharmaceuticals in *E. coli*, induced by a stable inorganic inducer. We reasoned that this would be useful in developing countries, where organic inducers might be unavailable or unduly expensive. Thus, we have developed a suite of vectors carrying promoters induced by the NarL transcription activator, which is triggered by nitrate ions.

**Figure 1:**
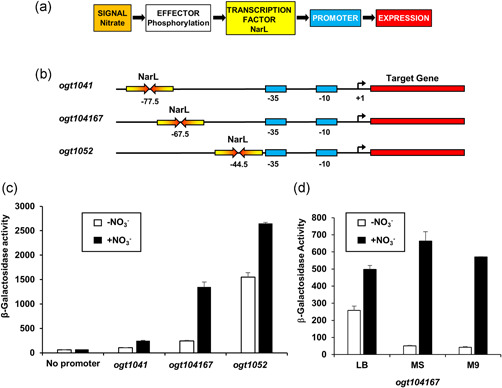
Expression analysis of the *ogt* promoter fragments used in this study. (a) Control of gene expression by nitrate and the NarL transcription activator protein. The presence of nitrate in the growth medium leads to the phosphorylation of the NarL transcription factor, enabling it to bind to target promoters and control transcript initiation by RNAP (Constantinidou et al., 2006; Darwin & Stewart, 1996; Santos‐Zavaleta et al., 2019; Stewart, 2003). (b) The panel shows schematic representations of the *ogt1041, ogt104167* and *ogt1052* promoter fragments. The NarL‐binding sites are shown as inverted arrows, −35 and −10 promoter elements are shown as rectangles, and transcript start sites (+1) are indicated by bent arrows. The location of each DNA site for NarL is labeled, according to convention, by the position of the center of the 7–2–7 sequence. Hence, position −77.5 is located between base pairs 77 and 78, upstream from the transcript start. At the *ogt1041*, *ogt104167*, and *ogt1052* promoters, the single DNA sites for NarL are located at positions −77.5, −66.5, and −44.5, respectively, and thus are 65, 55, and 32 bp, respectively, upstream from the corresponding promoter −10 element. (c) The panel shows measured β‐galactosidase activities in wild‐type JCB387 cells, carrying *ogt1041, ogt104167*, and *ogt1052* promoter fragments cloned into the pRW50 *lacZ* expression vector. Cells were grown in minimal salts media supplemented with 20 mM sodium nitrate, where indicated. (d) The panel shows measured β‐galactosidase activities in wild‐type JCB387 cells, carrying the *ogt104167* promoter fragment cloned into pRW50. Cells were grown in LB medium (LB), minimal salts media (MS), and M9 minimal medium (M9) supplemented with 20 mM sodium nitrate, where indicated. β‐Galactosidase activities are expressed as nmol ONPG hydrolyzed/min/mg dry cell mass and represent the average of three independent experiments. Error bars represent SD

The presence of nitrate in the growth environment triggers phosphorylation and activation of the *E. coli* NarL protein, resulting in its binding to at least 26 promoter regions, including 11 where it activates transcription (Figure 1a; Constantinidou et al., 2006; Darwin & Stewart, 1996; Santos‐Zavaleta et al., 2019; Stewart, 2003). Most bacterial transcription activators work by binding at their target and then making a direct contact with RNA polymerase (RNAP), which recruits and positions RNAP to the promoter region (Browning & Busby, 2016; Lee et al., 2012). Previous studies with NarL‐dependent promoters showed that activated NarL recognizes a 7‐base sequence element, and most targets consist of two copies of this element organized as an inverted repeat, separated by two base pairs (known as the “7‐2‐7” sequence; Darwin et al., 1997). Some NarL‐dependent promoters are particularly complicated, involving interactions with other transcription factors, for example, the *E. coli narG* promoter is coregulated by NarL and the anaerobically triggered transcription factor, FNR (Browning et al., 2010; Darwin & Stewart, 1996). However, we identified two promoter regions (*yeaR* and *ogt*) where NarL alone is able to activate transcript initiation (Ruanto et al., 2020; Squire et al., 2009). Here, we describe new derivatives of both the *ogt* and *narG* promoters, and use them to drive high‐level recombinant protein production (RPP), engineering them to optimize their activity and dependence on both NarL and nitrate.

## 2 MATERIALS AND METHODS

### 2.1 Bacterial strains, plasmids, and materials

*E. coli* K‐12 strains, plasmids, and promoter fragments used in this study are listed in Table S1 and oligonucleotide primers are in Table S2. Strains were grown in lysogeny broth (LB) (Sigma), Lennox broth (2% (w/v) peptone (Oxoid), 1% (w/v) yeast extract (Oxoid) and 170 mM NaCl), minimal salts medium (Squire et al., 2009) (minimal salts with 0.4% glycerol, 10% Lennox broth, 20 mM fumarate), or M9 minimal salts media (Sigma), supplemented with 0.36% glucose, 2 mM MgSO_4_, 0.1 mM CaCl_2_, all with appropriate antibiotic supplements (ampicillin 100 µg/ml and tetracycline 15 µg/ml).

### 2.2 Vector construction

To examine expression from each promoter construct, DNA fragments were initially cloned into the low‐copy number *lac* expression vector, pRW50 (Lodge et al., 1992). The *ogt1041*, *ogt104167*, and *ogt1052* promoters have been described previously (Ruanto et al., 2020). The NN series of promoters (i.e., *NN*(−81.5) to *NN*(−69.5)), which carry a consensus NarL 7–2–7 heptamer sequence upstream of a CRP binding site, centered at position −40.5, were constructed using PCR. Promoter fragments were generated using primers NN(−81.5) to NN(−69.5) with D10527 and pRW50/*CC*(−40.5) as a template. Purified PCR products were restricted with *EcoRI* and *HindIII* and cloned into pRW50. For the *narG CC*(−40.5) promoter, the full‐length *narG* promoter (*narG223*: DNA sequences from −223 to +70) was PCR amplified using primers narGup223 and narGDown, with *E. coli* K‐12 JCB387 genomic DNA as a template. In the next round of PCR, primers narGup223 and narGCC(−40.5) were used with pRW50/*narG223* to amplify the upstream region of the *narG* promoter. The resultant PCR product was then restricted with *EcoRI* and *BamHI* and cloned into pRW50/*CC*(−40.5) to generate the *narG CC*(−40.5) promoter. DNA substitutions were introduced into the promoter region of the *narG* CC(−40.5) promoter by PCR amplifying the downstream of *narG CC*(−40.5) with primers narGCC(TG) or narGCC(−10) and primer D10527. PCR products were cloned into pRW50/*narG CC*(−40.5) using *HindIII* and *BamHI* restriction sites. All constructs were verified by Sanger DNA sequencing.

For expression of recombinant proteins, the *ogt104167* and *narG CC*(−40.5) promoters were introduced into pET15b/*6his*‐*gfp* and pET20b (Novagen) plasmids. Promoters were PCR amplified using primer pairs ogt(*BglII*) and ogt(*XbaI*) or narG223(*BglII*) and CC(*XbaI*) with the relevant pRW50 construct. Purified PCR products were restricted with *BglII* and *XbaI* and cloned into pET15b/*6his*‐*gfp* and pET20b, replacing the canonical T7 RNA polymerase promoter. DNA, encoding hGH‐6His and anti‐IL‐1β‐6His scFv, from pHAK1 and pYU49 (Alanen et al., 2015; Matos et al., 2014), respectively, was cloned into pET20b/*ogt104167* and pET20b/*narG CC*(−40.5) using *Nde*I and *Sac*I (Figures S1 and S2). All constructs were verified by Sanger DNA sequencing. The pET22b vector constructs, which express hGH‐6His, and carry the *lac* O1O1, *lac* O3O1, and *tac* promoters (i.e., PAR1, PAR4L, and PAR8 constructs), were previously described by Hothersall et al. (2021) (Table S1).

### 2.3 Strain construction

The *ΔnarG s*train JCB387N11 was constructed using P1 transduction, by transferring the kanamycin resistance gene marker, from *E. coli* K‐12 strain BW25113 *narG::aph* into strain JCB387 (Thomason et al., 2007). Kanamycin‐resistant colonies were isolated and the presence of the *narG::aph* cassette was confirmed, using PCR with primers narGFw and narGRev. The kanamycin resistance cassette was then removed by transforming candidates with plasmid pCP20 (Cherepanov & Wackernagel, 1995).

### 2.4 β‐Galactosidase assays

pRW50 derivatives, containing *lacZ* promoter fusions, were transformed into the relevant *E. coli* K‐12 strains and β‐galactosidase activities were measured using a Miller protocol (Miller, 1972). Single colonies, carrying each construct, were inoculated into Lennox Broth and grown overnight at 37°C with shaking. To assay activities, overnight cultures were inoculated into 5 ml of minimal salts media and grown at 37°C with shaking until an OD_650_ = 0.5–0.6 (Squire et al., 2009). Where appropriate, the medium was supplemented with 20 mM sodium nitrate. To test the ability of household plant‐growth fertilizers to induce gene expression, solutions of BabyBio (SBM Life Science), Growmore (Westlands), Miracle‐Gro All Purpose and Miracle‐Gro LiquaFeed (Evergreen Garden Care) were added to a final concentration of 1% (v/v or w/v). β‐Galactosidase activities are expressed as nmol ONPG (*o*‐nitrophenyl‐β‐d‐galactopyranose) hydrolyzed/min/g dry cell mass and represent the average of three independent experiments.

### 2.5 Recombinant protein overexpression and detection

Cultures of *E. coli* strain JCB387N11, carrying pET expression plasmids containing the *ogt104167* and *narG CC*(−40.5) promoters and various target genes, were grown with shaking in 10 mL of minimal salts medium, until an OD_600_ = 0.3–0.5. Protein overexpression was induced by the addition of sodium nitrate and samples were taken after 3 h induction. To test the ability of household fertilizer to induce gene expression, BabyBio (SBM Life Science) was added to a final concentration of 1% (v/v). For pET22b constructs, which carry the *lac* O1O1, *lac* O3O1, and *tac* promoters (Hothersall et al., 2021), RPP was induced by the addition of IPTG to a final concentration of 1 mM. Anaerobic growth conditions were achieved by growing cultures statically in 100 ml minimal salts medium in 100 ml Duran bottles, as in our previous work (Filenko et al., 2007). Cultures were incubated at 37°C without shaking to OD_600_ = 0.3–0.5, and RPP was then induced by the addition of 20 mM sodium nitrate, with samples taken after 3 h induction.

Total protein samples were routinely prepared by resuspending normalized amounts of cells in 2× Laemmli loading buffer (Sigma), heating at 95°C for 3 min and centrifuging before loading. Normalized protein samples were resolved by reducing sodium dodecyl sulfate polyacrylamide gel electrophoresis (SDS‐PAGE) and analyzed using Coomassie blue staining and western blot analysis, as in our previous work (Browning et al., 2013). For western blot analysis, 6His‐GFP was detected using anti‐GFP antiserum raised in mouse (Sigma), and an anti‐mouse‐HRP secondary antibody (Sigma), hGH‐6His was detected using anti‐hGH antiserum raised in rabbit (Browning et al., 2019) and an anti‐rabbit‐HRP secondary antibody (Amersham), and anti‐IL‐1β‐6His scFv was detected using anti‐6His (C‐terminal)‐HRP (Invitrogen). Blots were developed using Pierce ECL western blotting analysis substrate and all gels and blots shown are representative experiments. To assess the aggregation of product in inclusion bodies, total, soluble, and insoluble protein samples were also prepared using an Agilent BugBuster, according to the manufacturer's instructions, as in our previous work (Hothersall et al., 2021).

### 2.6 Flow cytometry analysis

For flow cytometry analysis, 50 ml cultures in minimal salts medium were incubated with shaking at 30 or 37°C until the culture reached OD_600_ ~0.6 and then RPP was induced by the addition of 20 mM sodium nitrate. Cultures were analyzed using a BD Accuri C6 flow cytometer (Becton Dickinson). Samples were mixed with 0.2‐µm‐filtered PBS, and data were collected at a rate of 1000–4000 events per second, using slow flow and a forward scatter height (FSC‐H) threshold of 10,000 to eliminate noncellular material, until 20,000 events had been recorded per sample. Data were analyzed using CFlow software (BD). GFP fluorescence was detected using a 533/30 BP filter on channel FL1.

Live and dead cells were differentiated using propidium iodide (PI) (Wyre & Overton, 2014). The PI concentration used in the sample (final concentration of 4 μg/ml) and the gating to distinguish between live and dead cells was determined by measuring a mixture of live and dead cells. Dead cells were prepared by taking 2 ml of live cells pelleted by centrifugation at 13,000*g* for 1 min, washed in phosphate buffered saline (PBS), pelleted at 13,000*g* for 1 min, and resuspended in 1 ml of 70% ethanol for 5 min at room temperature. Ethanol was removed and the resulting material was suspended in PBS, pelleted at 13,000*g* and resuspended in 1 ml PBS. PI fluorescence was detected using a 670 LP filter on channel FL3.

## 3 RESULTS

### 3.1 Expression of recombinant protein using the *ogt104167* promoter

Our previous studies with the *ogt* promoter region showed that a single 7‐2‐7 DNA site for NarL is sufficient for NarL‐dependent induction of transcription when located 65, 55, or 32 base pairs (bp) upstream from the promoter −10 element, that is, the *ogt1041*, *ogt104167*, and *ogt1052* promoters, respectively (Figure 1b) (Ruanto et al., 2020). To examine expression from these promoters in more detail, each promoter fragment was cloned into the low copy number *lac* expression vector, pRW50, to generate *lacZ* transcriptional fusions that were transformed into the Δ*lac E. coli* K‐12 strain, JCB387. The expression of β‐galactosidase in JCB387, carrying each promoter, was then determined when cells were grown in the presence or absence of 20 mM sodium nitrate. Results in Figure 1c show that although *ogt1052* was a highly active promoter, it was poorly coupled to nitrate in the growth media. In contrast, the *ogt104167* and *ogt1041*, promoters were better coupled to nitrate levels, being more tightly regulated.

**Figure 2:**
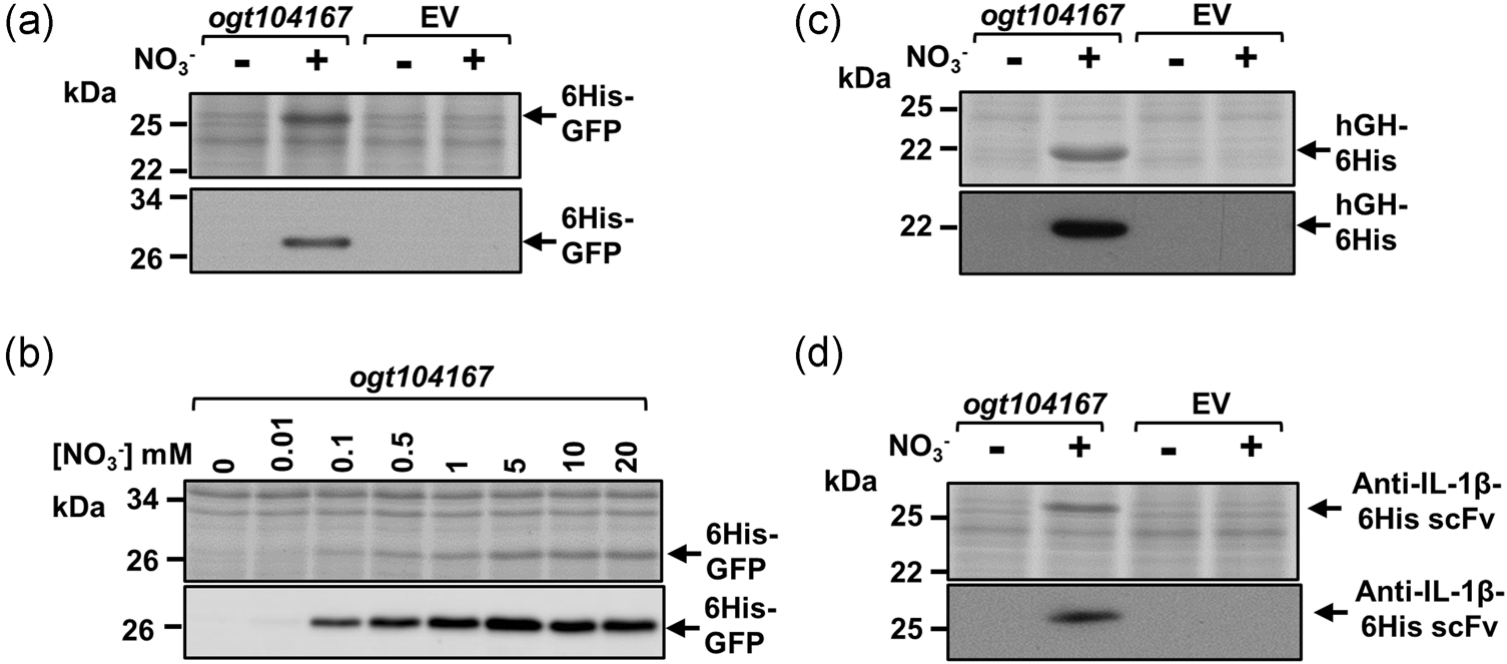
Recombinant protein production driven by the *ogt104167* promoter. The figure shows Coomassie blue‐stained sodium dodecyl sulfate polyacrylamide gel electrophoresis gels and western blots (below) that detail the expression of (a, b) recombinant 6His‐GFP, (c) hGH‐6His, and (d) anti‐IL‐1β‐6His scFv in *Escherichia coli* K‐12 JCB387N11 (Δ*narG*) cells grown in minimal salts medium after 3 h induction by the addition of 20 mM sodium nitrate (+) at an OD_600_ of 0.3–0.5. The DNA encoding each target was cloned into pET expression vectors carrying the *ogt104167* promoter fragment. Empty vector controls (EV) are included, where indicated.

As the *ogt104167* promoter was the more active, we chose this to examine heterologous gene expression. Therefore, the genes encoding His‐tagged green fluorescent protein (6His‐GFP), His‐tagged human growth hormone (hGH‐6His), and a His‐tagged variable fragment of a single‐chain antibody directed against interleukin 1β (anti‐IL‐1β‐6His scFv) (Figures S1 and 2) were cloned into pET vectors carrying *ogt104167*. As *ogt104167* was tightly controlled in minimal media (Figure 1d) expression was examined in minimal salts medium (Squire et al., 2009) and, to reduce the removal of nitrate inducer from the medium, we used an *E. coli* strain, lacking NarG, the major nitrate reductase. Results in Figure 2 illustrate SDS‐PAGE and western blot analysis of batch‐grown cultures in which RPP was induced for 3 h by the addition of 20 mM sodium nitrate. In each case, significant nitrate‐induced overexpression was seen, with little or no expression detected in the absence of nitrate. Furthermore, expression from *ogt104167* increased with increasing nitrate concentration, reaching a maximum at ~5 mM sodium nitrate (Figure 2b). Thus, the *ogt104167* promoter is a tightly regulated nitrate‐responsive promoter that can be used to drive expression of heterologous proteins in *E. coli*.

### 3.2 Construction and testing of the *narG CC*(−40.5) promoter

**Figure 3:**
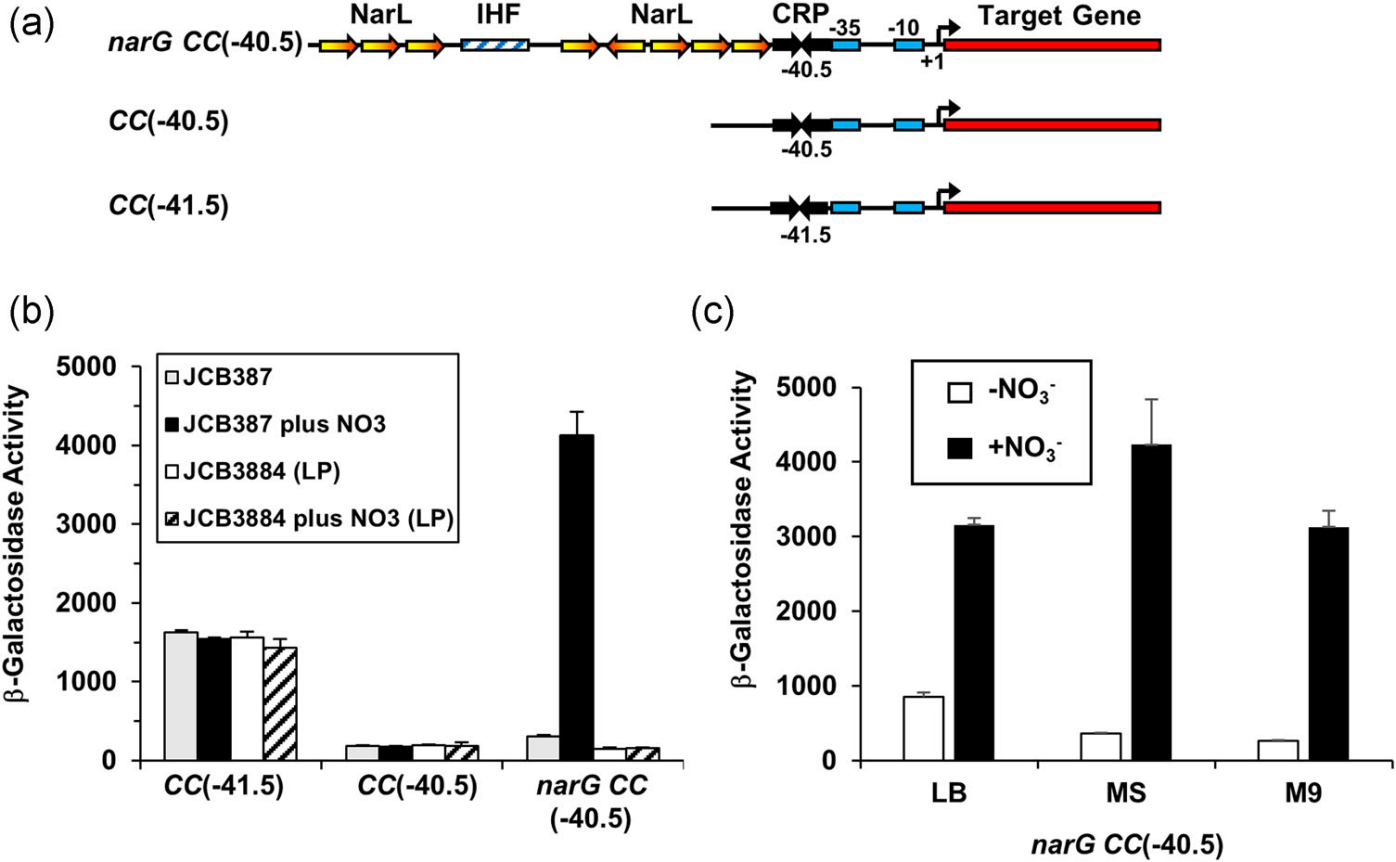
Expression analysis of the *narG CC*(−40.5) promoter. (a) The panel shows schematic representations of the *narG CC*(−40.5), *CC*(−40.5), and *CC*(−41.5) promoter fragments. The DNA sites for NarL and CRP are shown as arrows, the integration host factor (IHF)‐binding site is depicted as a hatched box and the promoter −35 and −10 elements shown as rectangles. Transcript start sites (+1) are indicated by bent arrows. (b) The panel shows measured β‐galactosidase activities in strain JCB387 and JCB3884 (*narL narP*) cells, carrying the *CC*(−41.5), *CC*(−40.5), and *narG CC*(−40.5) promoter fragments cloned into the pRW50 *lacZ* expression vector. Cells were grown in minimal salts media supplemented with 20 mM sodium nitrate (+NO_3_), where indicated. (c) The panel shows measured β‐galactosidase activities in JCB387 cells, carrying the *narG CC*(−40.5) promoter fragment cloned into pRW50, with cells grown in LB medium (LB), minimal salts media (MS), or M9 minimal medium (M9) supplemented with 20 mM sodium nitrate, as indicated. β‐galactosidase activities are expressed as nmol ONPG hydrolyzed/min/mg dry cell mass and represent the average of three independent experiments. Error bars represent SD.

Although controlled RPP was achieved with nitrate using *ogt104167*, we wanted to reach higher expression levels, while maintaining regulation by nitrate and NarL. Most bacterial transcription activators work by making a direct contact with RNAP, and this acts as molecular “velcro” to position the RNAP at the promoter (Browning & Busby, 2016; Finkelstein, 2005). At some promoters, different activators work together, with each factor contributing its contact to the “velcro” (Finkelstein, 2005; Lee et al., 2012). This is the case at the *E. coli narG* promoter where activity is co‐dependent on the binding of FNR and NarL (Walker & DeMoss, 1992, 1994). We decided to focus on developing a similar promoter, dependent on NarL and a second activator, but, as FNR functions only in anaerobic conditions, we used its homolog, CRP (the cyclic AMP receptor protein), that is active in most growth conditions (Li et al., 1998). Previous studies had shown that CRP (like FNR) activates transcription as a dimer, and activates optimally when the spacing between the center of the DNA site for CRP and the promoter −10 element is 29 base pairs (Gaston et al., 1988; Li et al., 1998; Rossiter et al., 2015). However, if the spacing is reduced to 28 base pairs, promoter activity falls to basal levels, but it can be restored by an upstream‐bound activator (Rossiter et al., 2015). Hence, starting with a simple semisynthetic CRP‐dependent promoter, *CC*(−41.5), carrying a single DNA site for CRP located 29 bp upstream from the promoter −10 element (Gaston et al., 1990; West et al., 1993), we first adjusted the spacing to 28 bp, and then sought to restore activity to the resulting promoter (*CC*(−40.5)) by inserting a single upstream 7–2–7 DNA site for NarL. However, despite trying a range of locations, we were unable to find a combination that resulted in nitrate‐regulated promoter activity (Figure S3). In contrast, when we inserted an upstream segment from the *narG* promoter covering the multiple DNA sites for NarL, the activity of the resulting promoter (*narG CC*(−40.5)) was massively induced by the inclusion of nitrate in the growth media. Figure 3 illustrates the different constructions (Figure 3a), together with assay data for each promoter, using our *lacZ*‐based expression vector (Figure 3b,c). To confirm that the observed activity of *narG CC*(−40.5) is due to a single promoter, co‐activated by NarL and CRP, we showed that nitrate‐dependent induction is dependent on NarL (Figure 3b) and that a single base substitution in the promoter −10 element reduces activity to basal levels (Figure S4). Additionally, introduction of CRP carrying the HL159 and KE101 substitutions, which prevent productive interactions between CRP and RNAP (West et al., 1993), also suppresses induction (Figure S4).

### 3.3 High‐level recombinant protein production using the *narG CC*(−40.5) promoter

**Figure 4:**
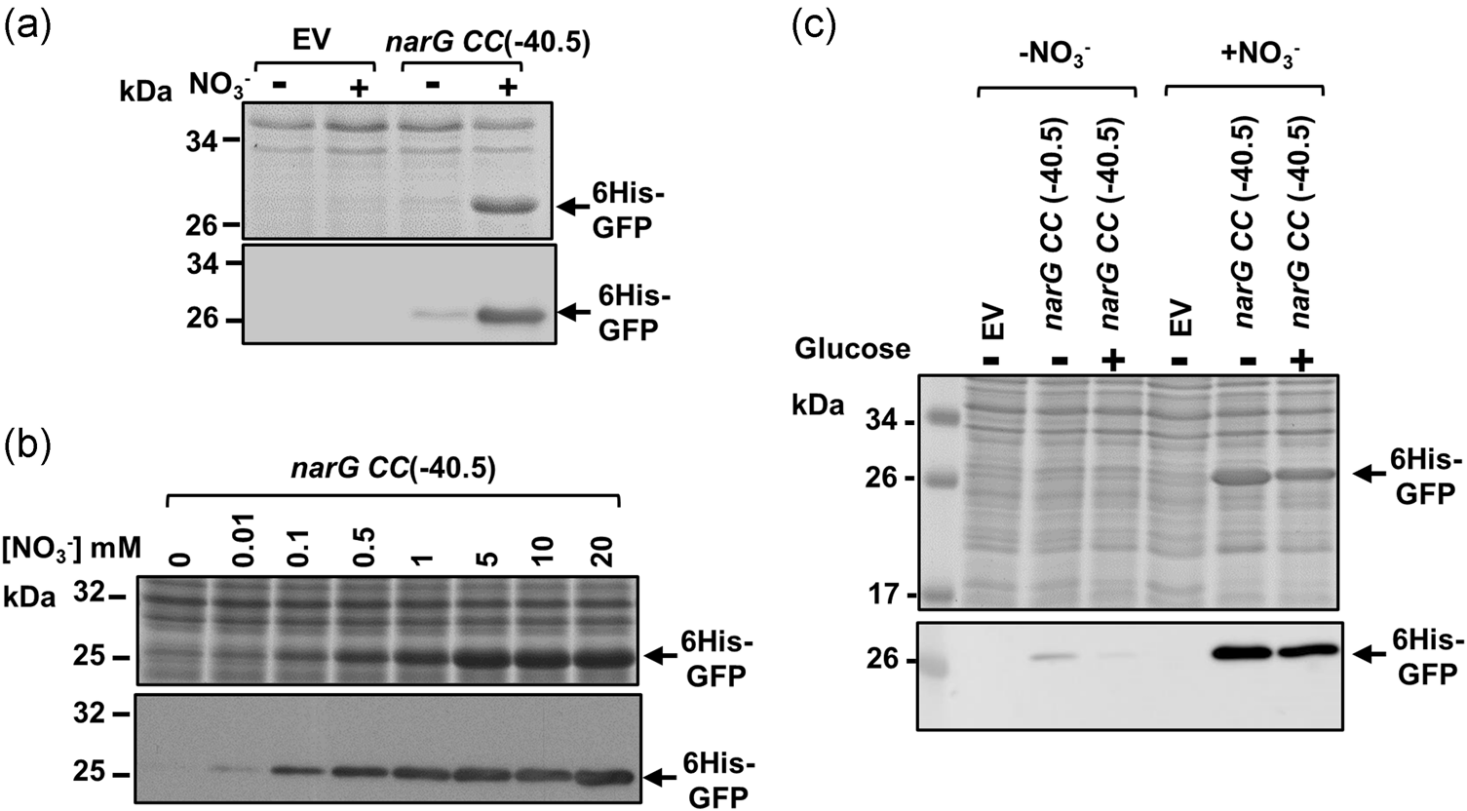
Expression of 6His‐GFP using the *narG CC*(−40.5) promoter. The figure shows Coomassie blue‐stained sodium dodecyl sulfate polyacrylamide gel electrophoresis gels and western blots (below) of JCB387N11 (Δ*narG*) cells expressing 6His‐GFP, using the *narG CC*(−40.5) promoter. The DNA encoding 6His‐GFP was cloned into pET expression vectors carrying the *narG CC*(−40.5) promoter fragment. Cells were grown in minimal salts media and RPP was initiated for 3 h (a, c) by the addition of 20 mM sodium nitrate or (b) by a range of sodium nitrate concentrations. In (c), cultures were supplemented with 0.4% glucose, where indicated. Empty vector controls (EV) were included.

**Figure 5:**
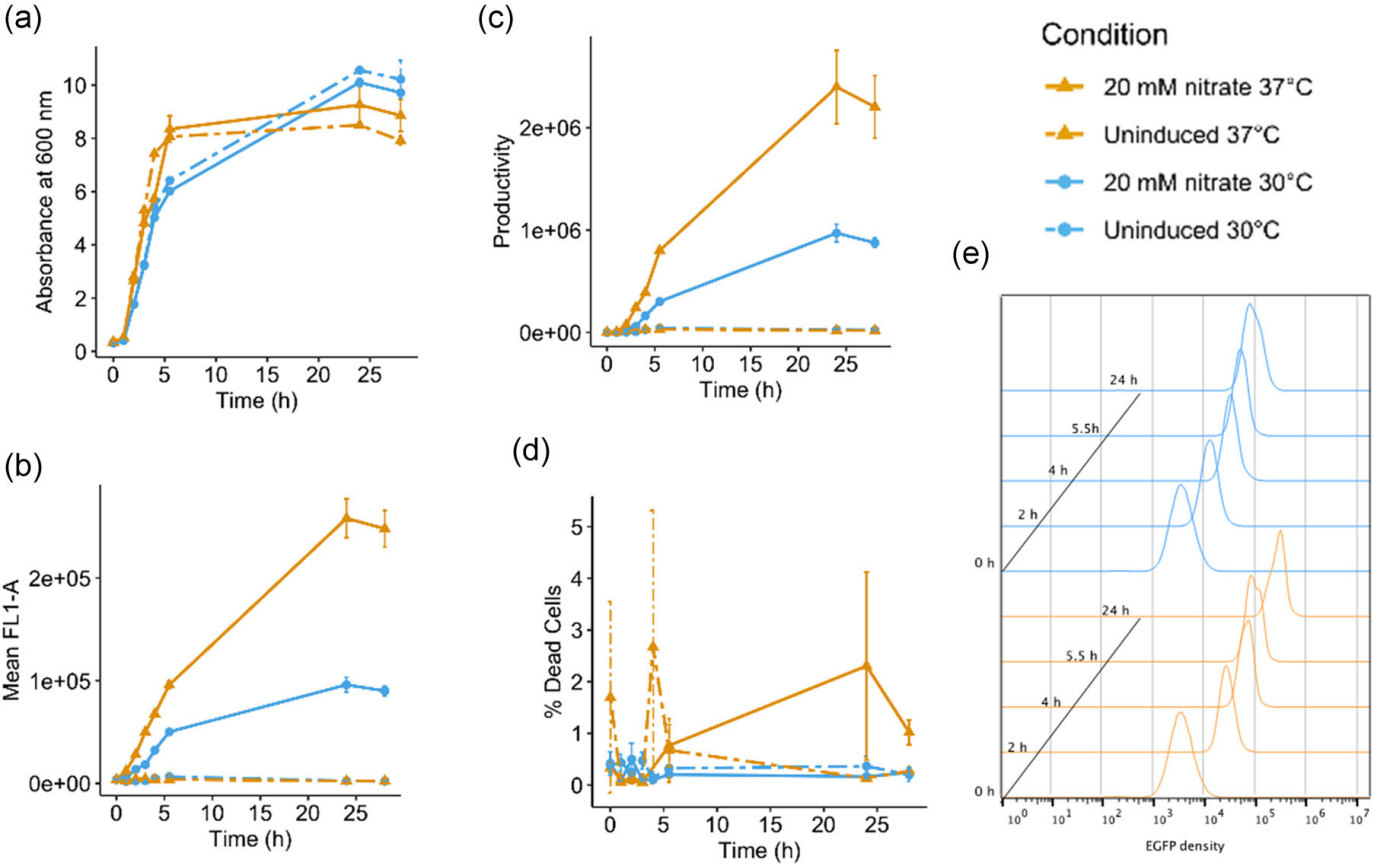
Analysis of 6His‐GFP expression from the *narG CC*(−40.5) promoter in *E. coli* JCB387N11 cells at 30 or 37°C. The figure shows the (a) growth, (b) mean GFP fluorescence measured by flow cytometry, (c) productivity (green fluorescence multiplied by OD_600_), and (d) percentage dead cells of 50 ml JCB387N11 (Δ*narG*) cultures expressing 6His‐GFP, using the *narG CC*(−40.5) promoter. Cells were grown in minimal salts media at either 30°C (blue) or 37°C (orange) for 25 h. Recombinant protein production was initiated by the addition of sodium nitrate to a final concentration of 20 mM, as cells reached an OD_600_ of ~0.6. Values represent the average values from duplicate flasks and error bars represent ±SD. (e) Flow cytometry analysis of green fluorescence from cells expressing 6His‐GFP grown and induced at either 30°C (blue) or 37°C (orange). Data are plotted as histograms showing the number of cells with different green fluorescence (FL1‐A) values.

The response of the *narG CC*(−40.5) promoter to nitrate suggested exploitation in overexpression vectors, and so it was introduced into a plasmid encoding 6His‐GFP. Results in Figure 4 show that, in batch cultures, 6His‐GFP was induced to high levels by nitrate and, as predicted, the *narG CC*(−40.5) promoter was stronger than *ogt104167* (Figure S5). Importantly, cell growth and viability was unaffected by nitrate‐induced 6His‐GFP expression, even at different temperatures (i.e., 30 and 37°C) for extended periods of time (Figure 5a–d). Analysis of individual cells by flow cytometry indicated that 6His‐GFP induction occurred homogeneously within the bacterial cell population (Figure 5e). Note that some low‐level expression of 6His‐GFP occurs in the absence of nitrate (Figure 4) but, because expression depends on CRP, it is subject to catabolite repression and can be suppressed by the inclusion of glucose in the growth medium (Figure 4c) (Hothersall et al., 2021; Kaur et al., 2018).

### 3.4 Expression of biopharmaceuticals using the *narG CC*(−40.5) promoter

**Figure 6:**
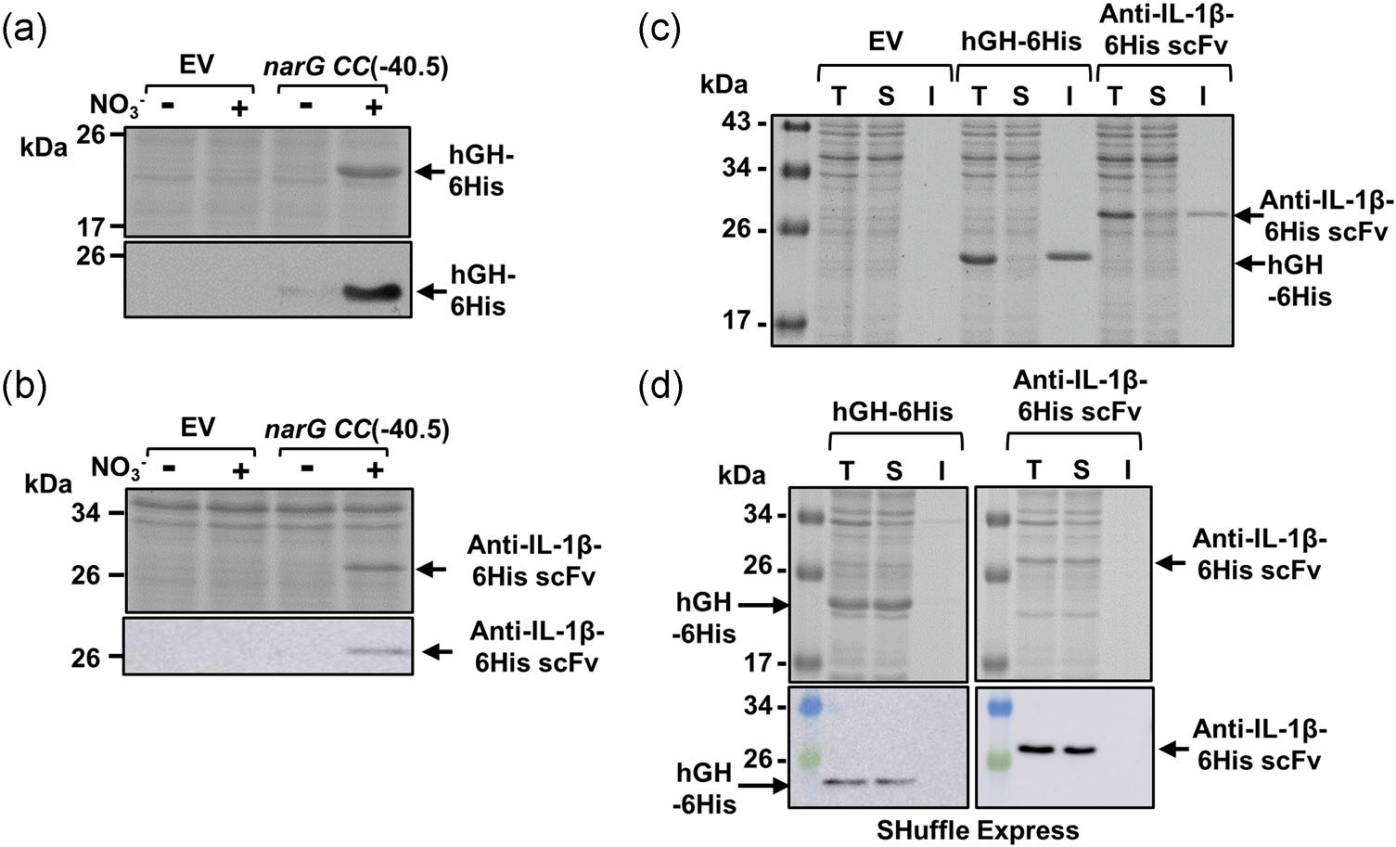
Solubility of recombinant hGH‐6His and anti‐IL‐1β‐6His scFv expressed in *Escherichia coli* JCB387N11 and SHuffle Express cells. (a, b)  Coomassie blue‐stained sodium dodecyl sulfate polyacrylamide gel electrophoresis (SDS‐PAGE) gels and western blots (below), which detail the expression of recombinant hGH‐6His and anti‐IL‐1β‐6His scFv, respectively, in *E. coli* K‐12 JCB387N11 (Δ*narG*) cells grown in minimal salts medium after 3 h induction by the addition of 20 mM sodium nitrate (+). (c,  d) Coomassie blue‐stained SDS‐PAGE gels investigating the solubility of hGH‐6His and anti‐IL‐1β‐6His scFv expressed in JCB387N11 (Δ*narG*) and SHuffle Express cells, respectively, using the *narG CC*(−40.5) promoter. Cultures were grown in minimal salts medium and protein production was induced by the addition of 20 mM sodium nitrate for 3 h. Harvested cells were lysed to prepare total (T), soluble (S), and insoluble (I) protein samples. In (d), a western blot (below) is included, showing the detection of hGH‐6His and anti‐IL‐1β‐6His scFv. Empty vector controls (EV) are indicated.

**Figure 7:**
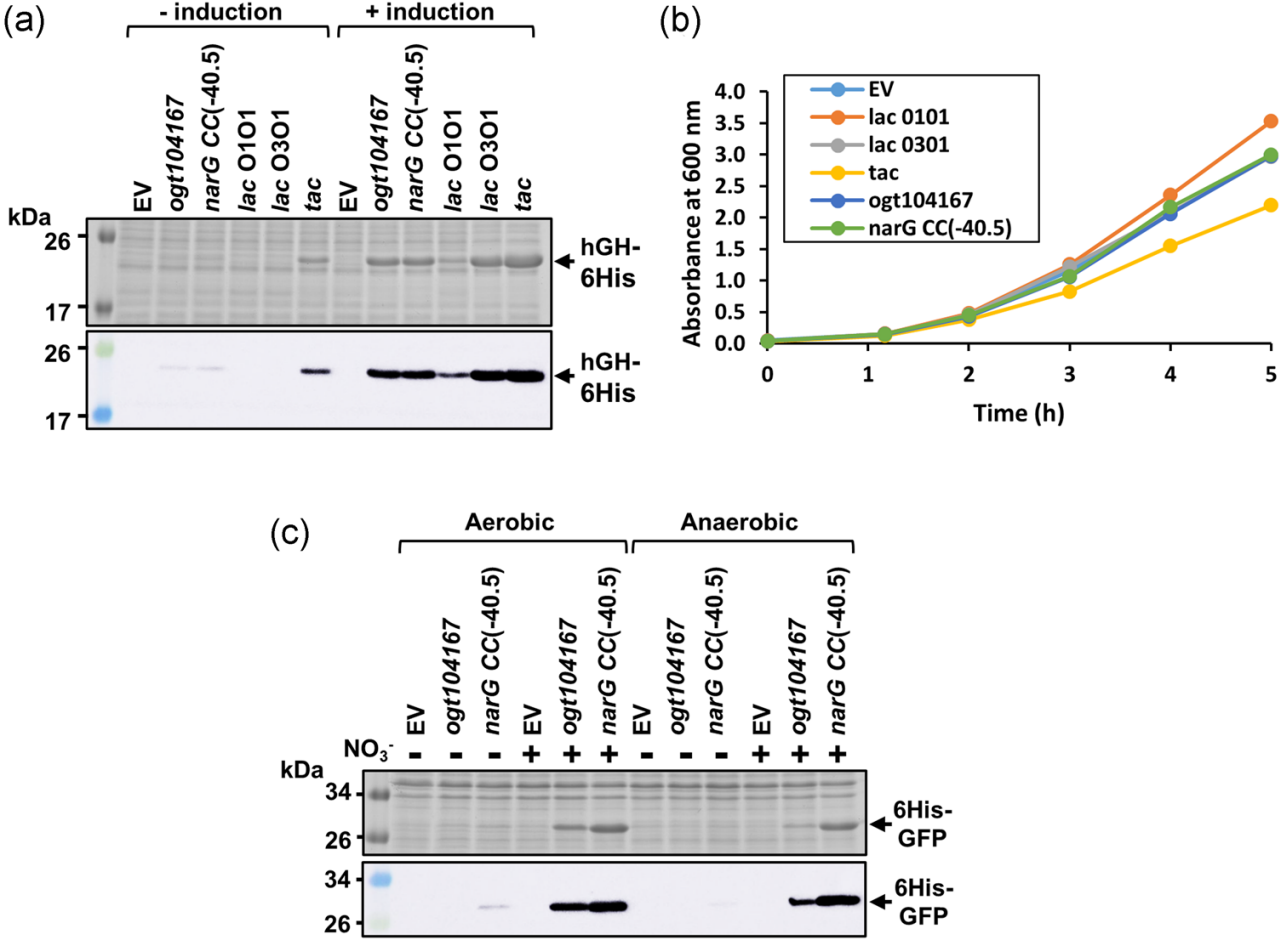
Comparison of protein expression from the *ogt104167* and *narG CC*(−40.5) promoters with *lac‐*based expression systems and different expression regimes. (a) Coomassie blue‐stained sodium dodecyl sulfate polyacrylamide (SDS‐PAGE) gel electrophoresis gel and western blot (below) that detail hGH‐6His expression and (b) growth profiles of induced cultures expressing hGH‐6His from *ogt104167* and *narG CC*(−40.5) in comparison to the low strength *lac* O1O1 promoter, the medium strength *lac* O3O1 promoter, and the strong *tac* promoter (Hothersall et al., 2021) in *E. coli* K‐12 JCB387N11 (Δ*narG*) cells. Cells were grown in minimal salts medium to an OD_600_ of 0.3–0.5. and protein production was induced for 3 h (+) by the addition of either 20 mM sodium nitrate to *ogt104167, narG CC*(−40.5), and the empty vector control (EV), or 1 mM IPTG to the *tac* and *lac*‐based vectors. (c) Coomassie blue‐stained SDS‐PAGE gel and western blot (below) that detail 6His‐GFP expression from *ogt104167* and *narG CC*(−40.5) under anaerobic and aerobic conditions. *E. coli* K‐12 JCB387N11 (Δ*narG*) cells were grown in minimal salts medium and protein production was induced for 3 h (+) by the addition of 20 mM sodium nitrate. Representative gels and growth curves are shown.

To examine expression from the *narG CC*(−40.5) promoter further, vectors were constructed to express hGH‐6His and the anti‐IL‐1β‐6His scFv. Like 6His‐GFP, expression of both hGH‐6His and the anti‐IL‐1β‐6His scFv was induced by nitrate (Figure 6a,b). Since the correct folding of hGH and scFv requires the formation of disulfide bonds, which is not favored in the reducing environment of *E. coli* cytoplasm, the majority of hGH‐6His and anti‐IL‐1β‐6His scFv was insoluble (Figure 6c). Hence, these targets were expressed at a lower temperature in *E. coli* SHuffle Express cells, a genetically modified *E. coli* strain that enables cytoplasmic disulfide bond formation. Under these conditions, hGH‐6His and the anti‐IL‐1β‐6His scFv were soluble (Figure 6d), highlighting the functionality of this expression system in different strains with different growth regimes. Furthermore, in the conditions that we have tried, RPP driven by the *narG CC*(−40.5) and *ogt104167* promoters is comparable to that of other promoters (e.g., the *tac* promoter and other *lac*‐based promoters; Hothersall et al., 2021), and has minimal effect on bacterial growth (Figure 7a,b).

## 4 DISCUSSION

Our overarching aim was to develop a robust regulated expression system for foreign proteins in *E. coli*. While decades of research have led to the discovery of scores of new regulators, very few have been exploited for biotechnology. Here, building on our previous work with NarL, we have developed two synthetic promoters whose activity is triggered by the addition of nitrate ions to the growth media. In previous reports, *E. coli narG* promoter derivatives have been exploited to drive RPP in anaerobic conditions (Hwang et al., 2017, 2018; Kim et al., 2011). Note that these previous studies focused on induction driven by anaerobiosis (dependent on FNR) rather than induction driven by nitrate (dependent on NarL). Here we have exploited the upstream sequences from the *E. coli narG* promoter to confer nitrate‐dependence on a core promoter cassette (dependent on CRP), thereby uncoupling nitrate‐dependent induction from anaerobic induction. This results in expression systems that can operate under both aerobic and anaerobic conditions (Figure 7c).

**Figure 8:**
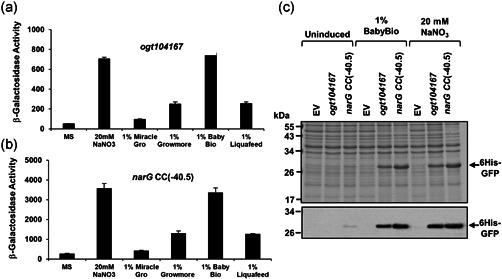
Induction of the *ogt104167* and *narG CC*(−40.5) promoters using garden fertilizers. The figure shows measured β‐galactosidase activities in wild‐type JCB387 cells, carrying either the (a) *ogt104167* or (b) the *narG CC*(−40.5) promoter fragments cloned into pRW50. Cells were grown in minimal salts media supplemented with 20 mM sodium nitrate or household fertilizers (Miracle‐Gro All Purpose, Growmore, BabyBio and Miracle‐Gro LiquaFeed) to a final concentration of 1% (v/v or w/v). β‐Galactosidase activities are expressed as nmol ONPG hydrolyzed/min/mg dry cell mass and represent the average of three independent experiments. Error bars represent SD. Panel (c) shows a Coomassie blue stained sodium dodecyl sulfate polyacrylamide gel electrophoresis gel and western blot (below), which detail the expression of recombinant 6His‐GFP in *E. coli* K‐12 JCB387N11 (Δ*narG*). Cells were grown in minimal salts medium and RPP was induced for 3 h by the addition of 20 mM sodium nitrate or 1% (v/v) BabyBio fertilizer. Empty vector controls (EV) are also included.

For many commercial expression systems, the inducer represents a significant cost. Since sodium nitrate costs less than one dollar per kilogram, we believe that the new vectors described here will be useful in locations where infrastructure is limiting. Additionally, since nitrate levels are high in many commercial fertilizers, recombinant protein can also be induced using inexpensive garden products available in local stores (Figure 8), and the facility to induce RPP without needing pure chemicals could prove useful in some situations. Thus, as well as cutting the cost of industrial RPP, our new promoters may have applications for protein production outside of the laboratory in the realm of DIY biology (Landrain et al., 2013).

## CONFLICT OF INTERESTS

The authors declare no conflict of interest.

## AUTHOR CONTRIBUTION

Joanne Hothersall, Tim W. Overton, Colin Robinson, Stephen J. W. Busby, and Douglas F. Browning devised the research program. Joanne Hothersall, Sandie Lai, Nan Zhang, Rita E. Godfrey, Patcharawarin Ruanto, Sarah Bischoff, and Douglas F. Browning performed the experiments.  Joanne Hothersall, Stephen J. W. Busby, and Douglas F. Browning wrote the manuscript with input from all the authors.

## Supporting information

Supplementary information.

## Data Availability

Data available in article supplementary material.
