# Supplementary material for: Inexpensive protein overexpression driven by the NarL transcription activator protein
Source: Biotechnol Bioeng. 2022 Mar 6;119(6):1614–23. doi: 10.1002/bit.28071 (PMC9314961; doi:10.1002/bit.28071)
Supplement: Supplementary file 1 — Supplementary information. [file BIT-119-1614-s001.docx]

***for Biotechnology and Bioengineering***  Edited by DFB on 10/2/2022

**Inexpensive protein overexpression driven by the NarL transcription activator protein.**

**Supplementary Material.**

Joanne Hothersall ^1^, Sandie Lai ^2,3^, Nan Zhang ^1^, Rita E. Godfrey ^1^, Patcharawarin Ruanto ^1^,

Sarah Bischoff ^4^, Colin Robinson ^4^, Tim W. Overton ^2^,

Stephen J. W. Busby ^1^*, Douglas F. Browning ^1,5^*.

^1^ Institute of Microbiology and Infection, School of Biosciences, University of Birmingham, Birmingham, B15 2TT, UK.

^2^ School of Chemical Engineering, University of Birmingham, Birmingham, B15 2TT, UK.

^3^ Present address: School of Life Sciences, University of Warwick, Gibbet Hill Campus, Coventry, CV4 7AL, UK.

^4^ School of Biosciences, University of Kent, Ingram Building, Canterbury, CT2 7NJ, UK

^5^ College of Health & Life Sciences, Aston University, Aston Triangle, Birmingham, B4 7ET, UK.

*To whom correspondence should be addressed:

Email: [s.j.w.busby@bham.ac.uk](mailto:s.j.w.busby@bham.ac.uk) Tel: +44 (0)121 414 5439

Email: [d.browning@aston.ac.uk](mailto:d.browning@aston.ac.uk)

**Supplementary Table S1. Strains, plasmids and promoter fragments used in this work.**

**Bacterial strains. Relevant genotype or description. Reference or source.**

BW25113 *E. coli* K-12 *lacI*^q^, *rrnBT14,* *∆lacZ*_WJ16_ ^1^

*hsdR514, ∆araBAD*_AH33,_ *∆rhaBAD*_LD78_.

BW25113 *narG::aph narG* replaced by a kanamycin resistance cassette. ^1^

M182 Δ*crp* Δ*crp* derivative of M182 (*E. coli* Δ*lac* K-12 strain) ^2^

JCB387 *E. coli* K-12 Δ*nir* Δ*lac*  ^3^

JCB3884 JCB387 Δ*narL* Δ*narP252*::*Tn1*0d(Cm) ^4^

JCB387N11 JCB387 *Δnir Δlac ΔnarG* This work.

SHuffle Express *F´ lac, pro, lacI^q^ / Δ(ara-leu)7697 araD139 fhuA2 lacZ::T7* NEB *gene1 Δ(phoA)PvuII phoR ahpC* galE (or U) galK λatt::pNEB3-r1-cDsbC (Spec^R^, lacI^q^) ΔtrxB rpsL150(Str^R^) Δgor Δ(malF) λ^-^, IN(rrnD-rrnE)1, rph-1*

**Bacterial plasmids.**

pRW50 A broad-host-range *lacZ* transcription fusion plasmid, (Tet^R^). ^5^

pET15b T7 RNA polymerase expression vector (Amp^R^). Novagen.

pET15b/ *6his-gfp* pET15b expressing 6His N-terminal GFP fusion. ^6^

pET20b T7 RNA polymerase expression vector (Amp^R^). Novagen.

pET22b T7 RNA polymerase expression vector (Amp^R^). Novagen.

Carries the *lacI* gene.

pET22b/ *lac* O1O1 pET22b carrying the weak *lac* O1O1 promoter (PAR1). ^6^

pET22b/ *lac* O3O1 pET22b carrying the medium strength *lac* O3O1 ^6^ promoter (PAR4L).

pET22b/ *tac*  pET22b carrying the strong *tac* promoter (PAR8). ^6^

pYU49 pET23 based vector with *ptac* promoter expressing ^7^

TorAsp anti-IL-1β-6His scFv.

pHAK1 pET23 based vector with *ptac* promoter expressing ^8^

TorAsp hGH-6His.

pCP20 Temperature sensitive recombineering plasmid, which ^9^

expresses the FLP recombinase (Amp^R^ and Cm^R^).

pDCRP A pBR322 derivative carrying the *crp* gene (Amp^R^). ^10^

pDCRP AR1^-^ A pDCRP derivative carrying a substitution in CRP activating ^10^

region AR1 (HL159).

pDCRP AR2^-^  A pDCRP derivative carrying a substitution in CRP activating ^10^

region AR2 (KE101).

pDCRP AR1^-^ & 2^-^ A pDCRP derivative carrying substitutions in CRP activating ^10^

regions AR1 (HL159) and AR2 (KE101).

**Promoters fragments. All fragments are flanked by EcoRI and HindIII sites.**

*ogt1041 E. coli ogt* promoter fragment carrying sequence from ^11^

-269 to +51 with a single consensus NarL 7-2-7 sequence

centered at position -77.5.

*ogt104167 E. coli ogt* promoter fragment carrying sequence from ^11^

-269 to +51 with a single consensus NarL 7-2-7 sequence

centered at position -67.5.

*ogt1052 E. coli ogt* promoter fragment carrying sequence from ^11^

-269 to +51 with a single consensus NarL 7-2-7 sequence

centered at position -44.5.

*CC*(-41.5) *E. coli melR* promoter derivative with a consensus DNA site ^12^

for CRP centred at position -41.5.

*CC*(-40.5) *E. coli melR* promoter derivative with consensus DNA site ^10^

for CRP centred at position -40.5.

*NN*(-81.5) *CC*(-40.5) carrying a consensus NarL 7-2-7 sequence centred This work. at position -81.5.

*NN*(-79.5) *CC*(-40.5) carrying a consensus NarL 7-2-7 sequence centred This work. at position -79.5.

*NN*(-77.5) *CC*(-40.5) carrying a consensus NarL 7-2-7 sequence centred This work. at position -77.5

*NN*(-75.5) *CC*(-40.5) carrying a consensus NarL 7-2-7 sequence centred This work. at position -75.5

*NN*(-73.5) *CC*(-40.5) carrying a consensus NarL 7-2-7 sequence centred This work. at position -73.5.

*NN*(-71.5) *CC*(-40.5) carrying a consensus NarL 7-2-7 sequence centred This work. at position -71.5.

*NN*(-69.5) *CC*(-40.5) carrying a consensus NarL 7-2-7 sequence centred This work. at position -69.5.

*narG223 E. coli narG* K-12 promoter fragment carrying sequences from This work

positions -223 to +70.

*narG CC*(-40.5) *CC*(-40.5) carrying the upstream DNA sequences from the This work. *E. coli narG* promoter.

**Supplementary Table S2. DNA Primers used in this work (all are shown 5' to 3').**

**Primer name. Sequence ^a^.**

D10520 CCCTGCGGTGCCCCTCAAG

D10527 GCAGGTCGTTGAACTGAGCCTGAAATTCAG

ogt(BglII) GGGGGAGATCTCAATCTGGTCGATTCTCGCCCCC

ogt(XbaI) GGGGGTCTAGAAATACCCGGACCTATCGGCAGACCGAC

NN(-81.5) GGGGGAATTCATATACCCATTAAGGAGTATATCGGTACCCGGGGATCA GGTAAATGTG

NN(-79.5) GGGGGAATTCATATACCCATTAAGGAGTATATGTACCCGGGGATCAGG TAAATGTGATG

NN(-77.5) GGGGGAATTCATATACCCATTAAGGAGTATATACCCGGGGATCAGGTA AATGTGATG

NN(-75.5) GGGGGAATTCATATACCCATTAAGGAGTATATCCGGGGATCAGGTAAA TGTGATGTAC

NN(-73.5) GGGGGAATTCATATACCCATTAAGGAGTATATGGGGATCAGGTAAATG TGATGTACATC

NN(-71.5) GGGGGAATTCATATACCCATTAAGGAGTATATGGATCAGGTAAATGTGA TGTACATC

NN(-69.5) GGGGGAATTCATATACCCATTAAGGAGTATATATCAGGTAAATGTGATG TACATCAC

narGup223 GGGGGAATTCCCATTAATATGTTACCCATGGGG

narGDown GGGGAAGCTTGGAATTTACTCATCGGTTTTCTCCTGTGGGAGC

narGCC(-40.5) GGGGGATCCATGTGATGTACATCACATTTAGATGGGGATGAAAAATAAA GTAAATTCC

narGCC(TG) GGGGGGGATCCCCCTCACTCCT**C**CCATAATTCTG

narGCC(-10) GGGGGGGATCCCCCTCACTCCTGCC**C**TAATTCTG

narG223(BglII) GGGGGAGATCTCCATTAATATGTTACCCATGGGGAATAC

CC(XbaI) GGGGGTCTAGAATGGCTCTCTTTCCTGGAATATCAG

narGFw TTTACAGTCTGTTATGTGGTGGCTG

narGRev GAAGCCCTGGCCCGGCTTGGTTTCC

**^a^** Restriction sites are underlined

**Supplementary Figure 1.**

**a *6his-gfp***

**TCTAGA**AATAATTTTGTTTAACTTTAAGAAGGAGATATACC**ATG**GGCAGCAGC**CATCATCATCATCATCAC**AGCAGCGGCCTGGTGCCGCGCGGCAGCCATATGGTGAGCAAGGGCGAGGAGCTGTTCACCGGGGTGGTGCCCATCCTGGTCGAGCTGGACGGCGACGTAAACGGCCACAAGTTCAGCGTGTCCGGCGAGGGCGAGGGCGATGCCACCTACGGCAAGCTGACCCTGAAGTTCATCTGCACCACCGGCAAGCTGCCCGTGCCCTGGCCCACCCTCGTGACCACCCTGACCTACGGCGTGCAGTGCTTCAGCCGCTACCCCGACCACATGAAGCAGCACGACTTCTTCAAGTCCGCCATGCCCGAAGGCTACGTCCAGGAGCGCACCATCTTCTTCAAGGACGACGGCAACTACAAGACCCGCGCCGAGGTGAAGTTCGAGGGCGACACCCTGGTGAACCGCATCGAGCTGAAGGGCATCGACTTCAAGGAGGACGGCAACATCCTGGGGCACAAGCTGGAGTACAACTACAACAGCCACAACGTCTATATCATGGCCGACAAGCAGAAGAACGGCATCAAGGTGAACTTCAAGATCCGCCACAACATCGAGGACGGCAGCGTGCAGCTCGCCGACCACTACCAGCAGAACACCCCCATCGGCGACGGCCCCGTGCTGCTGCCCGACAACCACTACCTGAGCACCCAGTCCGCCCTGAGCAAAGACCCCAACGAGAAGCGCGATCACATGGTCCTGCTGGAGTTCGTGACCGCCGCCGGGATCACTCTCGGCATGGACGAGCTGTACAAG**TAAGGATCC**

**b *hgh-6his***

**CATATG**TTCCCAACCATTCCCTTATCCAGGCTTTTTGACAACGCTATGCTCCGCGCCCATCGTCTGCACCAGCTGGCCTTTGACACCTACCAGGAGTTTGAAGAAGCCTATATCCCAAAGGAACAGAAGTATTCATTCCTGCAGAACCCCCAGACCTCCCTCTGTTTCTCAGAGTCTATTCCGACACCCTCCAACAGGGAGGAAACACAACAGAAATCCAACCTAGAGCTGCTCCGCATCTCCCTGCTGCTCATCCAGTCGTGGCTGGAGCCCGTGCAGTTCCTCAGGAGTGTCTTCGCCAACAGCCTGGTGTACGGCGCCTCTGACAGCAACGTCTATGACCTCCTAAAGGACCTAGAGGAAGGCATCCAAACGCTGATGGGGAGGCTGGAAGATGGCAGCCCCCGGACTGGGCAGATCTTCAAGCAGACCTACAGCAAGTTCGACACAAACTCACACAACGATGACGCACTACTCAAGAACTACGGGCTGCTCTACTGCTTCAGGAAGGACATGGACAAGGTCGAGACATTCCTGCGCATCGTGCAGTGCCGCTCTGTGGAGGGCAGCTGTGGCTTC**CATCATCATCATCATCACTAATAA**GGATCCGAATTC**GAGCTC**

**c anti-*IL-1β-6his scFv***

**CATATG**GATATCCAGATGACGCAGAGCCCGAGCAGCCTGAGCGCCAGCGTGGGTGACCGTGTGACCATTACCTGTCGTACCAGCGGCAACATTCATAACTATCTGACCTGGTACCAGCAGAAACCGGGCAAAGCGCCGCAGCTGCTGATTTATAATGCAAAAACCCTGGCAGATGGTGTGCCGAGCCGCTTTAGCGGCAGCGGTAGCGGTACCCAGTTCACCCTGACGATCAGCAGCCTGCAGCCGGAAGACTTTGCCAACTATTACTGCCAGCACTTCTGGAGCCTGCCGTTTACCTTCGGTCAGGGCACGAAAGTGGAAATTAAACGTACCGGCGGTGGCGGTAGCGGCGGTGGCGGTAGCGGCGGTGGCGGTAGCGGCGGTGGCGGTAGCGAGGTGCAGCTGGTTGAAAGCGGCGGTGGCCTGGTTCAGCCGGGTGGCAGCCTGCGTCTGAGCTGTGCGGCCAGCGGCTTTGATTTCAGCCGTTATGACATGAGCTGGGTGCGTCAGGCACCGGGTAAACGTCTGGAATGGGTTGCCTATATTAGCAGCGGTGGCGGTAGCACCTACTTTCCGGATACGGTGAAAGGCCGCTTCACCATCAGCCGTGACAACGCAAAAAATACGCTGTACCTGCAGATGAACAGCCTGCGCGCCGAAGATACCGCAGTTTATTACTGCGCCCGTCAGAATAAAAAACTGACGTGGTTCGACTACTGGGGTCAGGGCACGCTGGTTACGGTTAGCAGC**CATCATCATCATCATCACTAATAA**GGATCCGAATTC**GAGCTC**

**Supplementary Figure 1.** The DNA sequences of target proteins expressed in this study. The figure shows the DNA sequences of (a) *6his-gfp*, (b) *hgh-6his* and (c) anti-*IL-1β-6his scFv* used in this study. Restriction enzyme recognition sites (BamHI, NdeI, SacI and XbaI) used to clone each fragment into vectors are shown bold and underlined. For each construct the translation initiation codon (ATG) is green, the DNA encoding the 6His tag is purple and the translation stop codon (TAA) is red.

**Supplementary Figure 2.**

**a 6His-GFP Mw 29105 Da**

MGSS**HHHHHH**SSGLVPRGSHMVSKGEELFTGVVPILVELDGDVNGHKFSVSGEGEGDATYGKLTLKFICTTGKLPVPWPTLVTTLTYGVQCFSRYPDHMKQHDFFKSAMPEGYVQERTIFFKDDGNYKTRAEVKFEGDTLVNRIELKGIDFKEDGNILGHKLEYNYNSHNVYIMADKQKNGIKVNFKIRHNIEDGSVQLADHYQQNTPIGDGPVLLPDNHYLSTQSALSKDPNEKRDHMVLLEFVTAAGITLGMDELYK

**b hGH-6His Mw 23083 Da**

MFPTIPLSRLFDNAMLRAHRLHQLAFDTYQEFEEAYIPKEQKYSFLQNPQTSLCFSESIPTPSNREETQQKSNLELLRISLLLIQSWLEPVQFLRSVFANSLVYGASDSNVYDLLKDLEEGIQTLMGRLEDGSPRTGQIFKQTYSKFDTNSHNDDALLKNYGLLYCFRKDMDKVETFLRIVQCRSVEGSCGF**HHHHHH**

**c Anti-IL-1β-6His scFv Mw 27495 Da**

MDIQMTQSPSSLSASVGDRVTITCRTSGNIHNYLTWYQQKPGKAPQLLIYNAKTLADGVPSRFSGSGSGTQFTLTISSLQPEDFANYYCQHFWSLPFTFGQGTKVEIKRTGGGGSGGGGSGGGGSGGGGSEVQLVESGGGLVQPGGSLRLSCAASGFDFSRYDMSWVRQAPGKRLEWVAYISSGGGSTYFPDTVKGRFTISRDNAKNTLYLQMNSLRAEDTAVYYCARQNKKLTWFDYWGQGTLVTVSS**HHHHHH**

**Supplementary Figure 2.** The amino acid sequences of target proteins expressed in this study. The figure shows the amino acid sequences of the (a) 6His*-*GFP, (b) hGH-6His and (c) anti-IL-1β-6His scFv proteins used in this study. For each protein, the 6His tag is purple and predicted molecular weight (Mw) of each protein is given.

**
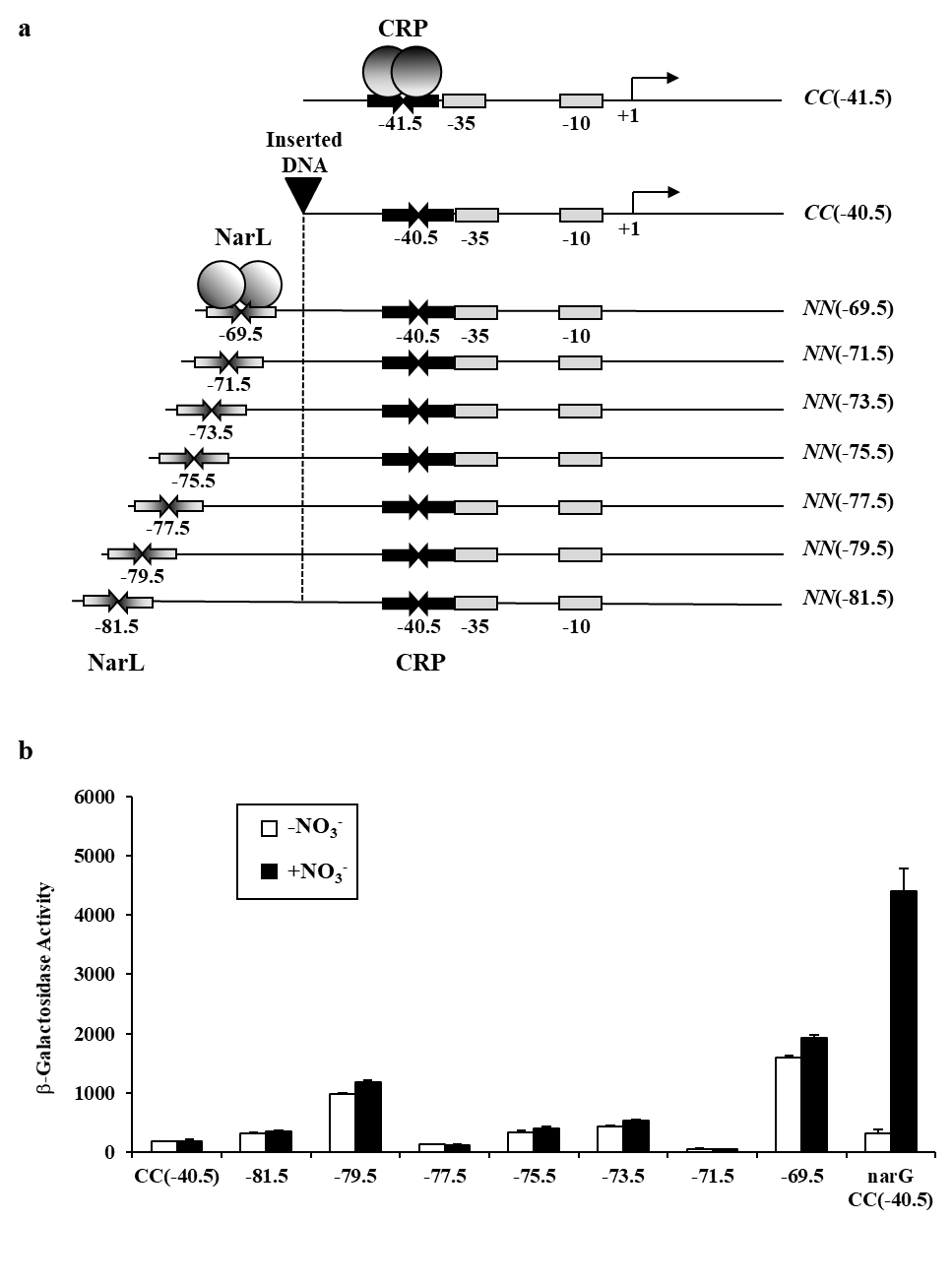
Supplementary Figure 3.**

**Supplementary Figure 3.** Expression analysis of the *NN* series of promoter fragments.

(a) The panel shows schematic representations of the *CC*(-41.5), *CC*(‑40.5) and the *NN* series of promoters (*NN*(-81.5) to *NN*(-69.5)). The DNA sites for NarL and CRP are shown as inverted horizontal arrows, -35 and ‑10 promoter elements are shown as rectangles and transcript start sites (+1) are indicated by a bent arrows. (b) The panel shows measured β-galactosidase activities in JCB387 cells, carrying either the promoter fragments in panel (a) or *narG CC*(-40.5), cloned into pRW50. Cells were grown in minimal salts media, supplemented with 20 mM sodium nitrate, as indicated. β-galactosidase activities are expressed as nmol ONPG (o-nitrophenyl-β-D-galactopyranose) hydrolysed min^-1^ mg^-1^ dry cell mass and represent the average of three independent experiments. Error bars represent standard deviation.

**Supplementary Figure 4.**

**
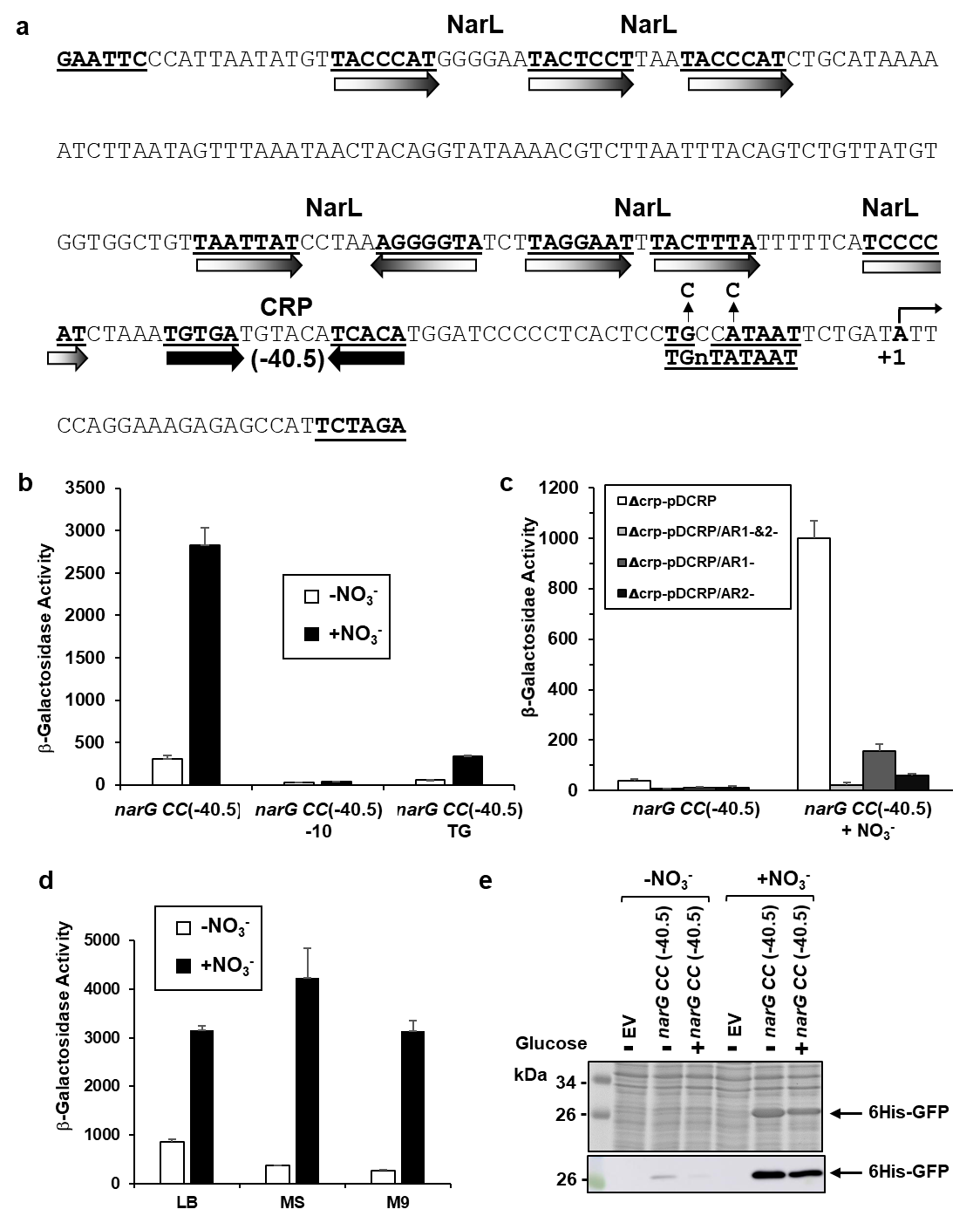
**

**Supplementary Figure 4.** Expression analysis of the *narG CC*(-40.5) promoter. (a) The panel shows the DNA sequence of the *narG CC*(‑40.5) promoter fragment. The NarL and CRP binding sites are shown as arrows, the transcript start site (+1) is indicated by a bent arrow and the -10 hexamer and extended -10 promoter element are in bold text and underlined, and aligned with the consensus sequence ^13^. The mutations introduced into the extended ­-­10 promoter element that disrupt the “TG” motif or the second base of the -10 hexamer are shown. (b) The panel shows measured β-galactosidase activities in JCB387 cells, carrying either the starting or mutant *narG CC*(‑40.5) promoter fragments, cloned into pRW50. (c) The panel shows measured β-galactosidase activities in M182 Δ*crp* cells, carrying *CC*(-40.5) and *narG CC*(-40.5) promoter fragments cloned into pRW50. Cells also carried either plasmid pDCRP or versions of pDCRP encoding substitutions in CRP activating regions AR1 (HL159) and/or AR2 (KE101). In (b) and (c) cells were grown in minimal salts media supplemented with 20 mM sodium nitrate, where indicated. β-galactosidase activities are expressed as nmol ONPG hydrolysed min^-1^ mg^-1^ dry cell mass and represent the average of three independent experiments. Error bars represent standard deviation.

**Supplementary Figure 5.**

**
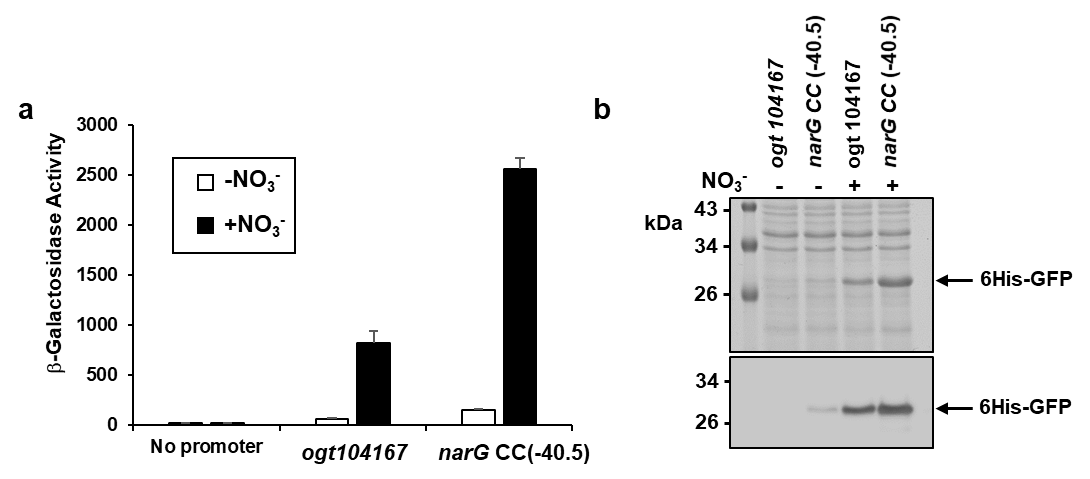
**

**Supplementary Figure 5.** Comparison of expression driven by the *ogt104167* and *narG CC*(‑40.5) promoters. (a) The panel shows measured β-galactosidase activities in wild-type JCB387 cells, carrying the *ogt104167* and *narG CC*(-40.5) promoter fragments cloned into pRW50. Cells were grown in minimal salts media supplemented with 20 mM sodium nitrate, where indicated. β-galactosidase activities are expressed as nmol ONPG hydrolysed min^-1^ mg^-1^ dry cell mass and represent the average of three independent experiments. Error bars represent standard deviation. (b) The panel shows a Coomassie blue stained SDS-PAGE gel and Western blot (below) of JCB387N11 (Δ*narG*) cells expressing 6His-GFP, using the *ogt104167* and *narG CC*(-40.5) promoters. Cells were grown in minimal salts media and RPP was initiated for 3 h by the addition of with 20 mM sodium nitrate, where indicated. In the Western blot, 6His-GFP was detected using mouse anti-GFP antibody.

**Supplementary References**

1. Baba, T. et al. Construction of *Escherichia coli* K-12 in-frame, single-gene knockout mutants: the Keio collection. *Molecular systems biology* **2**, 2006 0008 (2006).

2. Busby, S., Kotlarz, D. & Buc, H. Deletion mutagenesis of the Escherichia coli galactose operon promoter region. *Journal of molecular biology* **167**, 259-274 (1983).

3. Page, L., Griffiths, L. & Cole, J.A. Different physiological roles of two independent pathways for nitrite reduction to ammonia by enteric bacteria. *Archives of microbiology* **154**, 349-354 (1990).

4. Tyson, K.L., Cole, J.A. & Busby, S.J. Nitrite and nitrate regulation at the promoters of two *Escherichia coli* operons encoding nitrite reductase: identification of common target heptamers for both NarP- and NarL-dependent regulation. *Molecular microbiology* **13**, 1045-1055 (1994).

5. Lodge, J., Fear, J., Busby, S., Gunasekaran, P. & Kamini, N.R. Broad host range plasmids carrying the *Escherichia coli* lactose and galactose operons. *FEMS microbiology letters* **74**, 271-276 (1992).

6. Hothersall, J. et al. The PAR promoter expression system: Modified *lac* promoters for controlled recombinant protein production in *Escherichia coli*. *New Biotechnology* **64**, 1-8 (2021).

7. Matos, C.F. et al. Efficient export of prefolded, disulfide-bonded recombinant proteins to the periplasm by the Tat pathway in *Escherichia coli* CyDisCo strains. *Biotechnology progress* **30**, 281-290 (2014).

8. Alanen, H.I. et al. Efficient export of human growth hormone, interferon alpha2b and antibody fragments to the periplasm by the *Escherichia coli* Tat pathway in the absence of prior disulfide bond formation. *Biochimica et biophysica acta* **1853**, 756-763 (2015).

9. Cherepanov, P.P. & Wackernagel, W. Gene disruption in *Escherichia coli*: TcR and KmR cassettes with the option of Flp-catalyzed excision of the antibiotic-resistance determinant. *Gene* **158**, 9-14 (1995).

10. West, D. et al. Interactions between the Escherichia coli cyclic AMP receptor protein and RNA polymerase at class II promoters. *Molecular microbiology* **10**, 789-797 (1993).

11. Ruanto, P. et al. Activation by NarL at the *Escherichia coli ogt* promoter. *The Biochemical journal* **477**, 2807-2820 (2020).

12. Gaston, K., Bell, A., Kolb, A., Buc, H. & Busby, S. Stringent spacing requirements for transcription activation by CRP. *Cell* **62**, 733-743 (1990).

13. Browning, D.F. & Busby, S.J. Local and global regulation of transcription initiation in bacteria. *Nature reviews. Microbiology* **14**, 638-650 (2016).
